# Neural markers of error processing relate to task performance, but not to substance-related risks and problems and externalizing problems in adolescence and emerging adulthood

**DOI:** 10.1016/j.dcn.2024.101500

**Published:** 2024-12-24

**Authors:** Olga D. Boer, Thea Wiker, Shervin H. Bukhari, Rikka Kjelkenes, Clara M.F. Timpe, Irene Voldsbekk, Knut Skaug, Rune Boen, Valerie Karl, Torgeir Moberget, Lars T. Westlye, Ingmar H.A. Franken, Hanan El Marroun, Rene J. Huster, Christian K. Tamnes

**Affiliations:** a Department of Psychology, Education and Child Studies, Erasmus School of Social and Behavioral Science, Erasmus University Rotterdam, Rotterdam 3000 DR, Netherlands; b Department of Child and Adolescent Psychiatry, University Medical Center Rotterdam, Erasmus MC, Sophia Children's Hospital, Rotterdam 3000 CB, Netherlands; c PROMENTA Research Center, Department of Psychology, University of Oslo, Oslo, Norway; d Center for Precision Psychiatry, Division of Mental Health and Addiction, Oslo University Hospital & Institute of Clinical Medicine, University of Oslo, Oslo, Norway; e Department of Behavioural Science, Faculty of Health Sciences, Oslo Metropolitan University, OsloMet, Oslo, Norway; f Division of Mental Health and Substance Abuse, Diakonhjemmet Hospital, Oslo, Norway; g Department of Psychology, University of Oslo, Oslo, Norway; h Multimodal Imaging and Cognitive Control Lab, Department of Psychology, University of Oslo, Norway; i Department of Medical Genetics, Oslo University Hospital, Oslo, Norway; j KG Jebsen Center for Neurodevelopmental Disorders, University of Oslo, Norway; k Cognitive and Translational Neuroscience Cluster, Department of Psychology, University of Oslo, Norway

**Keywords:** Adolescence, EEG, ERP, Error processing, Externalizing behavior, Substance use

## Abstract

Detecting errors and adapting behavior accordingly constitutes an integral aspect of cognition. Previous studies have linked neural correlates of error processing (e.g., error-related negativity (ERN) and error-related positivity (Pe)) to task performance and broader behavioral constructs, but few studies examined how these associations manifest in adolescence. In this study, we examined neural error processing markers and their behavioral associations in an adolescent/emerging adult sample (*N* = 143, *M*_age_ = 18.0 years, range 11–25 years), employing a stop-signal task. Linear regressions were conducted using bootstrap resampling to explore associations between ERN/Pe peak amplitudes and latencies, stop accuracy, stop-signal reaction time (SSRT), and post-error slowing, as well as self-reported substance-related risks and problems and externalizing problems. After adjusting for age and sex, smaller frontocentral Pe amplitude and later Pe latency were associated with longer SSRT, and later Pe latency was associated with lower stop accuracy. This might indicate that the Pe, which is thought to reflect conscious error processing, reflects task performance on a response inhibition task better than the ERN, which reflects subconscious error processing. After correcting for multiple testing, there were no associations between ERN/Pe parameters and substance-related or externalizing problems, and no age interactions for these associations were detected.

## Highlights

• Smaller/later Pe peak related to lower task performance in a stop-signal task.
• Conscious (not subconscious) error processing may impact response inhibition.
• No relation between ERN/Pe and adolescent externalizing problems/substance use.

## Graphical Abstract

**Figure:**
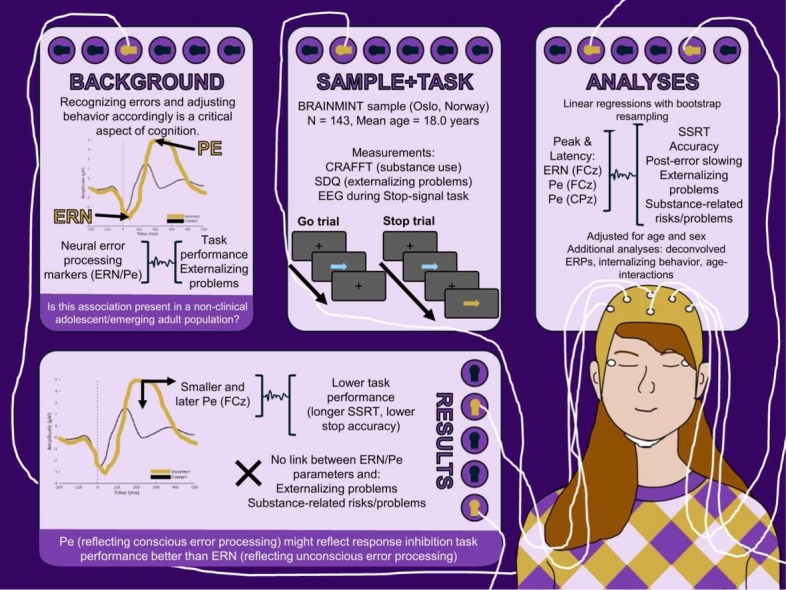

## 1 Introduction

Error processing constitutes an integral aspect of human cognition, and broadly refers to the ability to detect and react to mistakes and adjust behavior accordingly (Wessel, 2018). Deficient error processing has been proposed as a possible neurocognitive risk factor for psychopathology, such as externalizing problems (Luna et al., 2015, Ridderinkhof et al., 2004). Theoretically, individuals who are less capable of learning from mistakes and avoiding future mistakes, may be more at risk to develop externalizing problems. Although behavioral markers of error processing, such as error correction and speed-accuracy trade-off, provide important insights, neural markers offer additional sensitivity to the temporal dynamics of the decision making and error processing (Falkenstein et al., 2000). This added temporal resolution helps in understanding how errors are processed in real-time. Therefore, characterizing the dynamics and individual differences in neural markers of error processing has been a key interest within the cognitive and clinical neurosciences.

Two robust neurophysiological indices of error processing are the Error-Related Negativity (ERN) (Falkenstein et al., 1991, Gehring et al., 1993, Gehring et al., 2018) and the Error-Related Positivity (Pe) (Falkenstein, 1990, Falkenstein et al., 2000). Both can be detected using electroencephalography (EEG), and common parameters of interest are peak amplitude (i.e. the magnitude of the ERP waveform deflection) and peak latency (i.e. the time between the onset of the erroneous response and the peak amplitude). The ERN is a sharp negative deflection, peaking between 0 and 150 ms (ms) after an incorrect response (Coles et al., 2001), and is largest over frontocentral recording sites (Bush et al., 2000), reflecting activity of the anterior cingulate cortex (ACC), a brain region associated with error detection and conflict monitoring (Carter et al., 1998, Dehaene et al., 1994, Ullsperger and von Cramon, 2001). The ACC has been indicated as a source location for the ERN in both adults (Agam et al., 2011, Keil et al., 2010) and adolescents (Ladouceur et al., 2007), and it has been proposed that developmental changes in the ERN reflect structural and functional changes in the ACC (for a review, see Tamnes et al., 2013). The ERN is thought to reflect subconscious error processing, which can consist of conflict monitoring (Botvinick et al., 2001, Carter et al., 1998, Yeung et al., 2004) and/or reinforcement learning (Holroyd and Coles, 2002). The Pe, on the other hand, is a broader positive deflection peaking between 200 and 500 ms after an error. It initially seems to peak in frontocentral recording sites like the ERN, but can show a more centroparietal distribution from 300 to 500 ms post response (Ullsperger et al., 2014, Van Veen and Carter, 2002). Generally, the Pe is thought to reflect conscious awareness of an error (Endrass et al., 2007, Klein et al., 2013, Murphy et al., 2012, Nieuwenhuis et al., 2001, Wessel et al., 2011). Furthermore, Pe elicitation seems to be related to attentional processes required to adjust behavior following an error (Schroder et al., 2020).

ERN/Pe parameters may not directly reflect behavioral adaptation in a task, considering they reflect trial-specific monitoring processes, rather than behavioral adaptation across trials. Nevertheless, ERN/Pe amplitude and latency are often associated with task-related behavior (LoTemplio et al., 2023). In operationalizing error processing behavior, three common parameters are accuracy, reaction times and post-error slowing. While several previous studies have reported a relationship between increased accuracy and larger amplitudes (Falkenstein et al., 1995, Gehring et al., 1993, Maier et al., 2011) and shorter latencies of the ERN (Fiehler et al., 2005, Hoffmann and Falkenstein, 2010), others reported no significant association (Fiehler et al., 2005, Rodriguez-Fornells et al., 2002, Weinberg et al., 2010). When examining reaction times, special attention has been given to response inhibition latency, which is defined as the time required to suppress an already initiated response. This latency cannot be observed directly because successful inhibition implies the absence of an observable response. However, an estimation of this covert latency, known as the stop-signal reaction time (SSRT) can be calculated using a stop-signal task (Logan et al., 1984). While associations have been reported between SSRT and other ERP components, such as the failed stop N2 and P3 (Rueda-Delgado et al., 2021), to our knowledge, the direct association between ERN/Pe parameters and SSRT has not been studied specifically. Finally, post-error slowing (PES), the phenomenon where reaction times are slowed on trials directly following an error, is theorized to serve an adaptive goal of reducing error-likelihood on the next trial (Rabbitt, 1966), although it has also been interpreted as a maladaptive disruption of task performance (Ullsperger and Danielmeier, 2016, Wessel, 2018). Larger amplitudes of both ERN (for a review see LoTemplio et al., 2023) and Pe (Schroder et al., 2020) have been associated with greater PES, but null findings have also been reported (LoTemplio et al., 2023). Furthermore, meta-analytic effect sizes for the relationship between ERN and inter-individual PES were significant but small (Cavanagh and Shackman, 2015).

It has been suggested that ERN/Pe and task performance can reflect broader behavioral constructs implicated in attention-deficit/hyperactivity disorder (ADHD), conduct disorder (CD) and substance use disorder (SUD) (Ullsperger et al., 2014). Following the Hierarchical Taxonomy of Psychopathology (HiTOP), these disorders form an overarching disinhibited externalizing spectrum (Krueger et al., 2021). This classification is supported by findings of common genetic influences, environmental risk factors, childhood antecedents, cognitive abnormalities, neural alterations, and treatment response (Krueger et al., 2021) along with the high comorbidity among these externalizing disorders (Capusan et al., 2019, Giacobini et al., 2018, van Emmerik-van Oortmerssen et al., 2014). The link between ERN/Pe and externalizing problems might be explained by variations in ACC functioning, which potentially affect self-regulation and error monitoring and can lead to increased impulsivity (Shaw et al., 2011, Walhovd et al., 2012). Smaller ERN and Pe amplitudes have been associated with substance use dependence (Liu et al., 2023, Luijten et al., 2014, Zhang et al., 2021), behavioral addictions (Luijten et al., 2014), ADHD (Geburek et al., 2013, Kaiser et al., 2020) and other externalizing problems (Lutz et al., 2021, Lutz et al., 2021, Pasion and Barbosa, 2019, Vallet et al., 2021). Smaller Pe amplitudes were also reported in non-clinical populations, namely young, heavy but nondependent alcohol users as compared to a control group of light drinkers (Franken et al., 2017) and healthy students with inattention symptoms (Herrmann et al., 2009). Smaller ERN amplitudes at age 14 and 16 have also been prospectively associated with tobacco use initiation by age 18 (Anokhin and Golosheykin, 2016). Moreover, diminished ERN amplitudes have been observed in healthy individuals with a family history of SUD as compared to individuals without familial SUD (Euser et al., 2013, Riesel et al., 2019), suggesting a link between ERN amplitudes and substance use vulnerability. Although the link between ERN/Pe latencies and externalizing problems has been studied to a lesser extent, earlier ERN latencies have been associated with SUD (Chen et al., 2013) and ADHD (Chang et al., 2009), and another study suggested a link between delayed ERN latencies and behavioral disinhibition (Pasion et al., 2016). However, not all studies found a link between ERN/Pe latencies in SUD populations (see a meta-analysis by Liu et al., 2023) and ADHD and high impulsivity populations (Dimoska and Johnstone, 2007, Herrmann et al., 2009, McLoughlin et al., 2009, O'Connell et al., 2009, Ruchsow et al., 2005, Wiersema et al., 2009).

Substance use initiation before the age of 15 is a strong predictor of adult substance use and an increased risk for the development of SUD (DeWit et al., 2000, Trujillo et al., 2019). Moreover, adolescence is characterized by increased levels of risk-taking behavior (Spear, 2000), which might affect the interplay between substance use and other externalizing behaviors in youth. At the same time, both the ERN and Pe undergo developmental changes in childhood and adolescence, with the ERN increasing throughout adolescence, and the Pe increasing during childhood and stabilizing before adolescence (Boen et al., 2022, Overbye et al., 2019, Tamnes et al., 2013). However, while literature has indicated a link between ERN/Pe parameters and externalizing behavior when comparing clinical adolescent groups to healthy controls, fewer studies have focused on variations within the general population. Therefore, examining the interplay between ERN/Pe development and externalizing problems can provide valuable insights that could inform neurocognitive models of mental and behavioral disorders and possibly improve early detection or prevention efforts.

Taken together, while ERN/Pe show potential as biomarkers of externalizing problems, further examinations of the relationship between these electrophysiological indices and their assumed underlying behavioral constructs are needed. Moreover, as a first step in identifying populations at risk, the interplay between ERN/Pe and substance use and externalizing problems needs to be investigated in adolescent non-clinical samples. Therefore, in this study, our aim is twofold: first, we explore the relationship between ERN and Pe parameters and task behavioral parameters (stop accuracy, SSRT, and PES) in an adolescent and emerging adulthood sample (Brains and Minds in Transition (BRAINMINT) study, *N* = 143, age range 11–25 years). Second, we examine how ERN and Pe parameters are related to self-reported substance-related risks and problems and other externalizing problems. Then, through the addition of age interactions, we examine whether these associations vary depending on age. We hypothesize that smaller ERN/Pe amplitudes and larger latencies are associated with poorer task performance, as well as more externalizing problems, including substance-related risks and problems.

## 2 Methods

### 2.1 Sample

Brains and Minds in Transition (BRAINMINT) is an ongoing study based in Oslo, Norway. The study aim is to investigate the link between brain development and psychopathology risk in adolescence. To this end, the study utilizes magnetic resonance imaging (MRI), electroencephalography (EEG), cognitive data, questionnaires assessing mental health outcomes such as internalizing and externalizing symptoms and social and lifestyle factors, biomarkers, and genetics, with the possibility to link to Norwegian population and health registries. Participants were recruited through social media advertisements and collaborating studies. After completing an MRI research visit, which was conducted for other research purposes of the study, a subset of the participants was invited to participate in an EEG visit. Exclusion criteria therefore consisted of MRI contraindications. Participants completed various online questionnaires, including questionnaires assessing externalizing problems and substance-related risks and problems. Participants were considered for the current study if they completed an EEG measurement during the stop-signal task (N = 206) and questionnaires on externalizing and substance use behavior, leading to a total of N = 188 (18 participants with EEG data did not complete questionnaires). After applying task-specific exclusion criteria (see below), the final sample for analysis included 143 participants. Demographic information on the final sample is displayed in Table 1.

**Table 1:** Descriptive information of the study population (Total N = 143).

			Mean/N	SD/%	Distribution
**Demographic information**					
**Age (years)**			18.0	2.5	
Sex					
		Male	36	25.2 %	
		Female	107	74.8 %	
				
**CRAFFT (Car, Relax, Alone, Forget, Family/Friends, Trouble)**					
**Any substance use in the last 12 months**					
		Yes	89	62.2 %	
		No	54	37.8 %	
**Substance-specific: Any use in the last 12 months**					
Alcohol		Yes No	93 50	65.0 % 35.0 %	
Cannabis		Yes No	37 106	25.9 % 74.1 %	
Nicotine		Yes No	60 83	42.0 % 58.0 %	
Other substances		Yes No	19 124	13.3 % 86.7 %	
			
**CRAFFT sum score**			1.0	1.4	
			
**SDQ**					
**Externalizing sum score**			5.7	3.3	
Attention/hyperactivity scale			4.2	2.5	
Conduct scale			1.4	1.3	
			
			
**Internalizing sum score**			7.2	4.0	
Emotional problems scale			4.5	2.7	
Peer problems scale			2.6	1.9	

The study was approved by the Regional Committee for Medical and Health Research Ethics, South-East Norway. All participants, or their legal guardians in case of participants under the age of 16 years, provided informed consent prior to enrollment.

### 2.2 Stop signal task

The Stop Signal Task (SST) aims to measure inhibitory control, which is the ability to stop an ongoing action or behavior (Logan et al., 1984). A unique feature of the task is that it allows researchers to estimate response inhibition latency, which is the time an individual needs to inhibit a prepotent response. The current study used a visual version of the SST. The task was presented using a MS-7816 computer (Micro-Star International Co., Ltd, Taipei, Taiwan) with Windows 7. The participants were seated approximately 70 cm from the Benq xl2420t monitor (Benq Corp., Taipei, Taiwan) with a resolution of 1920 × 1080 and a refresh rate of 60 Hz. Stimuli were presented using the Psychophysics Toolbox (version 3.0.11; Brainard, 1997; Kleiner et al., 2007; Pelli, 1997) running in MATLAB R2014a (The MathWorks, Inc., Massachusetts, USA). Responses were given on a Cedrus RB-740 response pad (Cedrus corporation, San Pedro, CA, USA).

**Fig. 1:**
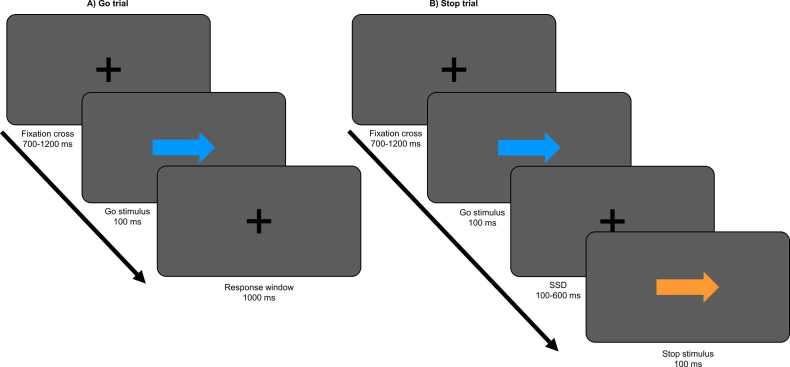
Depiction of the stop-signal task used in the current study. A) An example go-trial and B) an example stop trial. Color assignment of stimuli was counterbalanced.

In each trial (see Fig. 1), a black fixation cross centered on a grey background was presented with a duration between 700 and 1200 ms. Then, a ‘Go’ stimulus was presented for a fixed duration (100 ms), which was a blue arrow pointing either to the left or the right, followed by a 1000 ms response window. Participants were instructed to press the button on their right side with their right thumb when presented with a blue arrow pointing right, and vice versa (i.e. both thumbs were used). On a minority (24.4 %) of trials however, the ‘Go’ stimulus was followed by a ‘Stop’ stimulus (an orange arrow pointing in the same direction as the ‘Go’ stimulus), with a variable stop-signal delay (SSD). Participants were instructed to withhold from pressing any button when presented with a ‘Stop’ stimulus. The SSD was dynamically adjusted during the task to maintain a 50 % probability of successfully inhibiting the response on Stop trials: this was done by increasing SSD after a successful inhibition and decreasing SSD after an error. SSD was initiated as 250 ms, and could be adjusted with steps of 50 ms, down to a minimum of 100 and up to a maximum of 600 ms. Color assignment of stimuli was counterbalanced, meaning that the ‘Stop’ stimulus was orange for half of the participants and blue for the other half, and vice versa for the ‘Go’ stimulus.

Participants completed the task in a private room with minimal background noise. They first completed a practice block of 20 trials (consisting of 16 go trials and 4 stop trials) with feedback to get familiarized with the task. Afterwards, they completed 5 blocks of 90 trials each (consisting of 68 go trials and 22 stop trials), adding up to a total of 450 trials. Feedback to adjust speed and/or accuracy was provided after every block; if reaction time was over 600 ms on average, the message ‘be faster’ was displayed, if accuracy was below 40 %, the message ‘be more accurate’ was displayed.

Following recommendations of the recent stop-signal task consensus paper (Verbruggen et al., 2019), participants were considered for analysis if their ‘Stop’ trial accuracy was between 25 % and 75 %, their ‘Go’ trial accuracy was above 60 %, and if their average unsuccessful ‘Stop’ reaction time was shorter than their average ‘Go’ trial reaction time (i.e. following the horse race model, Band et al. 2003). Values outside these thresholds might reflect overcautious response styles, difficulties understanding the task, or lack of motivation to complete the task and might distort the data if not excluded. Applying these recommendations lead to exclusion of 10 participants.

Stop accuracy, go accuracy, SSRT, and post-error slowing were computed. Stop accuracy was defined as the number of successful stops (i.e., the absence of a response following a ‘Stop’ signal) divided by the total number of ‘Stop’ trials, expressed as a percentage. Go accuracy was defined as the number of correct responses (i.e. responses that are in time and in the indicated direction (left/right)) on ‘Go’ trials, divided by the total number of ‘Go’ trials, expressed as a percentage. SSRT was operationalized using the integration method, which involves subtracting the mean SSD from the ‘Go’ reaction time distribution percentile corresponding to the probability of a ‘Stop’ error (Band et al., 2003, Logan et al., 1984). This calculation can be displayed as follows: SSRT = RT_p(respond|signal)*N_ -SSD, where p(respond|signal) is the probability of responding when a stop signal is presented, N is the total number of Go trials, and RT_p(respond|signal)*N_ is the reaction time that corresponds to position p(respond|signal)*N in the distribution of Go RTs. Post-error slowing was operationalized using the ‘robust’ method (Dutilh et al., 2012), meaning the mean reaction time of trials prior to an unsuccessful ‘Stop’ trial were subtracted from the mean reaction time of trials following an unsuccessful stop trial. If errors occurred consecutively, the post-error RT of the first error and the pre-error RT of the last error were excluded from the post-error and pre-error means, respectively.

### 2.3 EEG data acquisition and processing

EEG was recorded using a BioSemi Active-Two amplifier system in ActiView version 8.0 (BioSemi, The Netherlands) on a Dell Precision 7530 laptop until mid-January 2023 and on an ASUS expert center D900MC_D900SC computer (ASUSTek Computer INC., Taipei, Taiwan) with Windows 10. EEG was recorded from 64 scalp sites (10–20 system) with Ag/AgCl electrodes that were placed in an elastic cap. Additionally, two electrodes were attached to the two outer canthi of both eyes (HEOG), two were attached to the abductor pollicis brevis (APB) muscle of the left and right thumb to measure muscle activity (EMG), two were attached to the fingerprint region of the index and middle finger of the nondominant hand to measure skin conductance, and a heart rate measure involved one electrode attached to the right collarbone/clavicle, and one attached to the left hip bone/anterior iliac crest. Finally, participants wore a respiration belt that was connected separately to the BioSemi amplifier. The eight external electrodes and the respiration belt are not part of the current analyses and the following description of the data processing only applies to the 64 scalp electrodes.

**Fig. 2:**
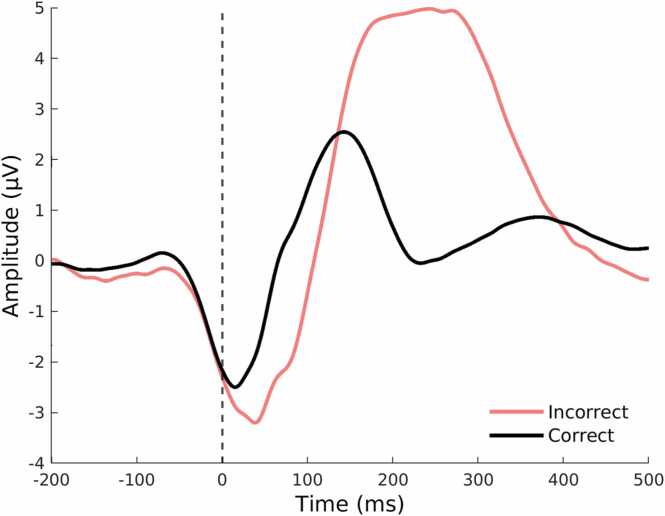
Grand average response-locked (response at time = 0 ms) event-related potentials for correct (black) and incorrect (red) responses in a stop-signal task, at FCz of all participants (N = 143). The waveform after incorrect (red) responses is characterized by a sharp negative peak at M = 65.7 ms (ERN), and a broader positive peak at M = 296.3 ms (frontocentral Pe). The waveform after correct (black) responses does not show these ERPs, but a correct response negativity (CRN) occurs due to stop-signal task characteristics.

EEGLAB (version 2022.0) was used for data preprocessing (Delorme and Makeig, 2004). Offline, the EEG signal was filtered with a 40 Hz low-pass filter (Kaiser window), then down sampled to 512 Hz and filtered with a 0.1 Hz high-pass filter (Clayson et al., 2021). Bad channels (with a channel criterion of a correlation of less than.6 between a given channel and its estimate based on other channels, and a line noise criterion of 4 SDs) were interpolated using spherical interpolation. Furthermore, the data was re-referenced using average referencing. Independent component analysis (ICA) was used for artifact rejection. Using ICLabel, components that showed more than 35 % chance of being eye movement/blinks and more than 50 % chance reflecting muscle activity were rejected (*M* = 3.7, range = 0–23). For the stop-signal task, data were segmented in epochs of 1300 ms: 500 ms before and 800 ms after a response, and the time period of 300–100 ms before the response was used for baseline correction. This time window was chosen because the onset of the ERN can precede the response (Clayson et al., 2021), reflecting motor preparation and other anticipatory processes (see Fig. 2). Finally, epochs that contained an amplitude larger than 100 μV (indicative of noise) were rejected. On average, 35 epochs (range = 0–198) were rejected per participant. Participant EEG data was considered for analysis if it contained at least 20 analyzable error-epochs, which led to 27 additional exclusions after applying task-based exclusions.

**Fig. 3:**
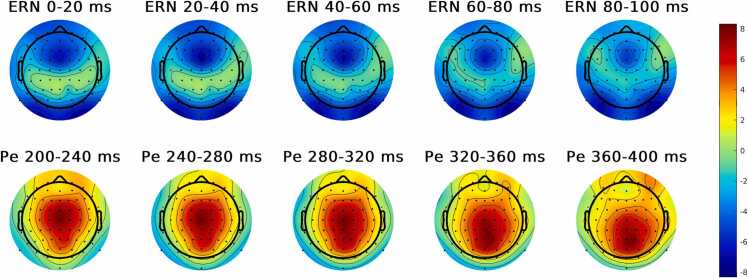
Topographic distribution of EEG amplitude (μV) averaged within various time bins following error responses across individuals (N = 143). The ERN, peaking between 0 and 100 ms after an incorrect response was largest over frontocentral recording sites (specifically, FCz). The Pe, peaking between 200 and 400 ms after an error, primarily peaked in frontocentral recording sites (FCz), but shows a more centroparietal distribution from 300 to 400 ms post response*.*

ERN and Pe were defined as the peak amplitude in the time-windows 0–150 ms and 200–500 ms after onset of an unsuccessful stop (error) response, respectively. To reduce the impact of random fluctuations, peak amplitudes were extracted by averaging the 5 data points (9.8 ms) around the minimum (ERN) or maximum (Pe) amplitude in the specified time window. Topographic heatmaps were used to visualize at which channel location ERN and Pe showed the largest peak amplitudes (see Fig. 3). For ERN, FCz was used as the channel location of interest. Pe was subdivided into a frontocentral Pe (measured at FCz) and a centroparietal Pe (measured at CPz) (Van Veen and Carter, 2002). Error-related ERPs were based on an average number of 38.4 trials (SD = 9.0, range = 20–56). ERP average waveforms for correct and incorrect responses are displayed in Fig. 2. The ERP average waveforms show an early negative deflection (albeit smaller in amplitude and earlier than the ERN) in correct responses as well, which is called the correct response negativity (CRN) (Vidal et al., 2000). The CRN amplitude is thought to reflect uncertainty in the response correctness (Scheffers and Coles, 2000) and/or effortful vigilance (Matsuhashi et al., 2021, Van Noordt et al., 2016, van Noordt et al., 2015). In a stop-signal task, CRN amplitude might be higher than in other tasks, considering there is a need for monitoring after the correct response to ensure an inhibition was not required (i.e., a ‘Stop’ signal did not appear), necessitating more focused attention (Van Noordt et al., 2016, van Noordt et al., 2015).

### 2.4 Substance-related risks and problems

The CRAFFT+N questionnaire is a screening tool used to identify substance-related risks and problems among adolescents. Compared to the original CRAFFT (Car, Relax, Alone, Forget, Family/Friends, Trouble) (Knight et al., 1999) that focuses on alcohol, cannabis, and other drug use, the CRAFFT+N contains an additional question to assess nicotine/tobacco use (Wheeler et al., 2004). The questionnaire has shown high sensitivity (.61–1.00) and specificity (.33–.97) in identifying substance use problems in adolescents in various (non)clinical settings (Dhalla et al., 2011, Knight et al., 2003, Mitchell et al., 2014, Skogen et al., 2013).

Participants completed the questionnaire online. The first four questions assessed the number of days a participant had used a certain substance (alcohol, cannabis, nicotine, other drugs) in the past twelve months. If the participants’ response was at least 1 in one of these four questions, they completed the following six questions: “Have you ever ridden in a car driven by someone (including yourself) who was ‘high’ or had been using alcohol or drugs?”; “Do you ever use alcohol or drugs to relax, feel better about yourself, or fit in?”; “Do you ever use alcohol or drugs while you are by yourself, or alone?”; “Do you ever forget things you did while using alcohol or drugs?”; “Do your family or friends ever tell you that you should cut down on your drinking or drug use?” and “Have you ever gotten into trouble while you were using alcohol or drugs?”. If the participant responded 0 to the first four questions, only the first question (car) was completed after. The CRAFFT score was composed of the total amount of yes-responses on the final six questions (ranging from 0 to 6), with a higher score indicating more substance use and related problems.

### 2.5 Externalizing problems

Externalizing problems were assessed using the Strengths and Difficulties Questionnaire (SDQ) (Goodman, 1997). This was done by combining the subscales conduct problems (e.g., “Often fights with other young people or bullies them”) and hyperactivity/inattention (e.g., “Restless, overactive, cannot stay still for long”), resulting in a total of 10 items. Items were scored on a 3-point scale (0 = not true, 1 = somewhat true, 2 = certainly true) based on self-report of the participant. Sum scores ranged from 0 to 20, with higher scores indicating more externalizing problems. The SDQ has shown satisfactory internal consistency and test-retest reliability with Cronbach’s alpha ranging from.70–.81 (Essau et al., 2012, Goodman, 2001) for the total SDQ,.66–.73 for the hyperactivity/inattention subscale and.41–.67 for the conduct problems subscale. Although all subscales can be examined separately, a factor model that combined the hyperactivity/inattention and conduct problems subscales into one externalizing factor has shown good fit for multiple samples (Essau et al., 2012). Therefore, the externalizing sum score was used in the current study.

### 2.6 Statistical analyses

First, we obtained study sample characteristics for descriptive purposes. Considering the right-skewness of the CRAFFT sum score distribution and leptokurtic distribution of stop accuracy, we used non-parametric bootstrap linear regression models with 1000 samples. This approach involves resampling with replacement from the original dataset to create multiple bootstrap samples (Efron and Tibshirani, 1994). Each sample is then used to construct a linear regression model, resulting in a distribution of regression coefficients and associated statistics that account for the uncertainty introduced by non-normal data. In the primary analyses, we first investigated the link between peak ERN and Pe amplitudes and latencies and the following SST behavioral measures: stop accuracy, SSRT, and post-error slowing. Then, we investigated the association between peak ERN and Pe amplitudes and latencies and an externalizing sum score, as well as the CRAFFT score. For ERN, we focused on the FCz channel location, and for Pe, we used the FCz and CPz channel locations separately (see Fig. 3). Models were fitted both with and without covariate adjustment for sex (Fischer et al., 2016) and age at EEG measurement (Boen et al., 2022, Overbye et al., 2019, Tamnes et al., 2013), and a false discovery rate (FDR) correction was applied to control for Type I errors (Benjamini and Hochberg, 1995).

Several secondary analyses were performed. First, as stop-signal task data consists of closely spaced events (e.g., stop-signal and response), deconvolution was performed as an additional analysis to account for the potential overlap of neural responses and to isolate event-specific neural activity (see Supplemental Figure 1). To this end, we used the Unfold 1.2 - EEG Deconvolution Toolbox for MATLAB (Ehinger and Dimigen, 2019). Deconvolution was not possible for one participant because the quality of their EEG data fell below the threshold required for the deconvolution, due to its specific sensitivity to artifacts. This resulted in a sample size of N = 142 for further analyses involving deconvolved ERP parameters.

Second, as stop accuracy is influenced by dynamic adjustment of the SSD, and is therefore complex to interpret, we reran analyses with go accuracy as the indicator for task accuracy. Third, we included an internalizing sum score as an additional covariate, as the ERN amplitude seems to have a dissociable pattern across psychopathology: while reduced ERN amplitudes are associated with externalizing disorders, increased ERN amplitudes have been associated with internalizing disorders (Moser et al., 2013). This sum score was formed by combining the subscales emotional problems and peer problems of the SDQ (Goodman, 1997). Next, to investigate whether any associations varied depending on age, we reran analyses with additional interactions between age and ERP parameters. Finally, to investigate whether ERP parameters were associated with any reported substance use, rather than substance-related risks and problems, we ran logistic regressions for reported alcohol use, cannabis use, nicotine use and other substance use separately. Originally operationalized as the number of days of use in the last 12 months, we dichotomized these variables (use in the last 12 months vs. no use in the last 12 months) due to right-skewness of the data.

All analyses were performed in R, version 4.3.2. The following packages were used: Dplyr 1.1.4, ggplot2 3.5.1, boot 1.3–31, corrplot 0.94, RColorBrewer 1.1–3, viridisLite 0.4.2, tidyr 1.3.1 and magrittr 2.0.3.

## 3 Results

**Fig. 4:**
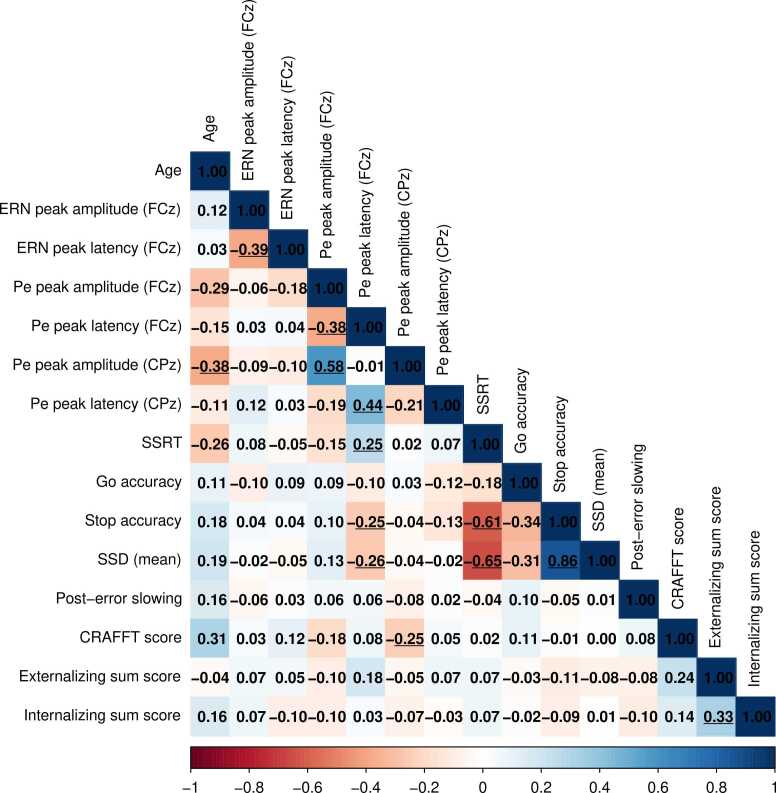
Pearson’s correlation matrix of age, ERP parameters, behavioral parameters, substance-related risks and problems, externalizing and internalizing behavior. Significant (p < .05) correlations are underlined. Abbreviations: ERN = error-related negativity, Pe = error-related positivity, SSRT = stop-signal reaction time, SSD = stop-signal delay.

Sample descriptive (N = 143) is displayed in Table 1 and Table 2. A correlation matrix of all variables of interest is displayed in Fig. 4. Briefly, 74.8 % were female and 62.2 % reported any substance use in the last 12 months. Participants who reported to have used alcohol, cannabis, nicotine or other substances, reported a median of 14 days of alcohol use, 3 days of cannabis use, 5 days of nicotine use and 3 days of other substance use in the last 12 months, respectively.

**Table 2:** Descriptive statistics of ERP parameters and task parameters in the current study (N = 143).

	M (min, max)	SD
***ERP parameters***		
ERN peak (µV)	−10.90 (−23.32, −3.37)	3.81
ERN latency (ms)	65.65 (31.38, 95.89)	12.52
Pe peak (FCz) (µV)	14.51 (2.27, 34.77)	5.64
Pe latency (FCz) (ms)	296.3 (233.32, 388.96)	28.54
Pe peak (CPz) (µV)	13.36 (2.82, 27.87)	4.95
Pe latency (CPz) (ms)	321.1 (241.14, 402.70)	31.32
***Task parameters***		
RT (go) (ms)	567.3 (374.49, 779.18)	85.55
RT (unsuccessful stop) (ms)	483.1 (332.81, 656.07)	69.26
Go accuracy (%)	94.22 (71.76, 100.00)	5.37
Stop accuracy (%)	52.59 (27.27, 73.64)	6.30
SSRT (ms)	193.6 (84.24, 336.10)	39.85
SSD (ms)	361.8 (122.67, 582.75)	99.88
Post-Error Slowing (ms)	60.34 (−82.53, 222.34)	48.66

Participants who were excluded due to task-related criteria (N = 45) did not differ from the included participants (N = 143) in substance-related risks and problems, externalizing behavior or ERP parameters, but did show shorter SSRT (*t*(77.28) = 2.85, *p* < .01), higher stop accuracy (*t*(51.36) = -4.00, *p* < .001) and lower go accuracy (*t*(49.17) = 4.35, *p* < .001).

### 3.1 Correlations between variables of interest

As expected, SSRT and stop accuracy showed a significant negative correlation (*r*(141) = -.61, p < .001), as high stop accuracy and low SSRT both indicate better task performance. Moreover, SSD correlated highly with SSRT (*r*(141) = .65, *p* < .001) and stop accuracy (*r*(141) = .86, *p* < .001), as both variables are directly related to SSD. These correlations, as well as the significant correlation between SSRT and stop accuracy (*p* < .001), are expected as these variables are based on reaction times and accuracy from the same task. Correlations between post-error slowing and SSRT/stop accuracy were small, which supports that post-error slowing can serve both an adaptive (i.e. increasing task performance) and maladaptive function (i.e. decreasing task performance) (Rabbitt, 1966, Ullsperger and Danielmeier, 2016). Age correlations were generally in the expected direction, although not significant: older participants seemed to have a lower SSRT, higher stop accuracy, and higher CRAFFT score. Older participants also showed lower centroparietal Pe peak amplitudes r(141) = -.38, *p* = .01). Peak amplitude and peak latency were significantly negatively correlated for ERN (*r*(141) = -.39, *p* < .01) and Pe (*r*(141) = -.38, *p* = .02), meaning a stronger ERN correlated with a later peak latency, and a stronger Pe correlated with an earlier peak latency. The correlation between ERN peak amplitude and Pe peak amplitude was small (*r*(141) = -.06), although in the expected direction, i.e., a more negative ERN peak with a larger Pe peak. Finally, the externalizing sum score showed positive correlations with both the CRAFFT score (*r*(141) = .24) and the internalizing sum score (*r*(141) = .33, *p* = .04).

### 3.2 ERN/Pe parameters and stop-signal task outcomes

After correcting for age and sex, reduced (β = −1.80, bootstrap 95 % CI [-2.83, −0.59], *SE* =.58, *t*(139) = -3.10, *p* < .01, *p*_FDR_ = .04) and delayed frontocentral Pe peak amplitudes (β =.31, bootstrap 95 % CI [.06,.54], *SE* =.11, *t*(139) = 2.75, *p* = .01, *p*_FDR_ = .04) were associated with a longer SSRT, indicating a slower response inhibition process. Furthermore, delayed frontocentral Pe peak latency was associated with lower stop accuracy, indicating an increased percentage of errors on stop trials (β = −.05, bootstrap 95 % CI [-.10, −.02], *SE* =.02, *t*(139) = -2.86, *p* < .01, *p*_FDR_ = .04). No associations were observed between ERN/centroparietal Pe parameters and task performance parameters (SSRT, stop accuracy and post-error slowing).

### 3.3 ERN/Pe parameters and substance-related risks and problems and other externalizing problems

In models without covariate adjustment (β =.02, bootstrap 95 % CI [.01,.04], *SE* =.01, *t*(141) = 2.23, *p* = .03, *p*_FDR_ = .14) as well as in models with adjustment for age and sex (β =.02, bootstrap 95 % CI [.01,.04], *SE* =.01, *t*(139) = 2.17, *p* = .03, *p*_FDR_ = .16), an initial association between delayed Pe peak latency and more externalizing behavior did not survive an FDR multiple testing correction. Similarly, smaller frontocentral as well as centroparietal Pe peak amplitude was associated with a higher CRAFFT score (indicating more substance related risks and problems) in models without covariate adjustment, before, but not after multiple testing correction (β = −.04, bootstrap 95 % CI [-.08, −.01], *SE* =.02, *t*(141) = -2.22, *p* = .03, *p*_FDR_ = .14; β = -.07, bootstrap 95 % CI [-.17,.40], *SE* = .02, *t*(141) = -3.02, *p* = .003, *p*_FDR_ = .06). No other associations were observed between ERN/Pe parameters and substance-related risks and problems and externalizing behavior.

### 3.4 Secondary analyses

First, we reran analyses after performing deconvolution on the EEG signal, accounting for the potential overlap of neural responses. Similar to the original analyses, smaller frontocentral Pe peak amplitude was associated with both longer SSRT (β = −1.74, bootstrap 95 % CI [-10.19, 9.91], *SE* =.73, *t*(138) = -2.34, *p* = .02, *p*_FDR_ = .39, after controlling for age and sex) and higher CRAFFT score (unadjusted: β = −.07, bootstrap 95 % CI [-.05,.47], *SE* =.02, *t*(138) = -2.84, *p* = .005, *p*_FDR_ = .10; adjusted for age and sex: β = -.05, bootstrap 95 % CI [-.08,.53], *SE* = .03, *t*(138) = -1.99, *p* = .048, *p*_FDR_ = .48), although the associations were not significant after FDR correction. As in the original analyses, ERN parameters were not associated with task performance nor substance-related risks and externalizing problems.

Second, we reran analyses with go accuracy as the indicator of task accuracy. Both in models with and without covariate adjustment, ERN/Pe parameters were not associated with go accuracy, indicating no relationship between ERN and Pe and the occurrence of omission errors or wrong-side responses.

Third, models were additionally adjusted for internalizing behavior. Although results were consistent with findings of the main analysis before multiple testing correction, no associations remained after an FDR correction (*p*_FDR_’s = .05).

Fourth, to examine potential age-related variations in the association between ERP parameters and behavior, we included age-interaction terms in additional regression models. In models without covariate adjustment (β = −.73, bootstrap 95 % CI [-1.27, −.10], *SE* =.29, *t*(140) = -2.50, *p* = .01, *p*_FDR_ = .27; β = -1.04, bootstrap 95 % CI [-1.70, −.42], *SE* = .35, *t*(140) = -2.97, *p* = .003, *p*_FDR_ = .10) as well as in models with adjustment for age and sex (β = −.75, bootstrap 95 % CI [-1.39, −.15], *SE* =.29, *t*(138) = -2.60, *p* = .01, *p*_FDR_ = .21; β = -1.06, bootstrap 95 % CI [-1.72, −.50], *SE* = .35, *t*(140) = -3.04, *p* = .003, *p*_FDR_ = .08), there seemed to be a negative age interaction for the relationship between frontocentral and centroparietal Pe peak amplitude and post-error slowing. This would imply that as age increases, there is a decrease in the association between a larger Pe peak and more post-error slowing. Age interactions were also observed before multiple testing correction for the relationship between centroparietal Pe peak latency and stop accuracy (β = −.02, bootstrap 95 % CI [-.04, −.01], *SE* =.01, *t*(138) = -2.61, *p* = .01, *p*_FDR_ = .10), SSRT (β =.09, bootstrap 95 % CI [.01,.17], *SE* =.04, *t*(138) = 2.07, *p* = .04, *p*_FDR_ = .24) and externalizing problems (β =.01, bootstrap 95 % CI [.001,.02], *SE* =.004, *t*(138) = 2.39, *p* = .02, *p*_FDR_ = .12). Nonetheless, none of the age interaction remained after FDR correction.

Finally, we ran logistic regression models on the relationship between ERN/Pe parameters and any reported substance use in the last 12 months (yes/no), as well as any alcohol, cannabis, tobacco and other substance use separately. Without covariate adjustment, a smaller frontocentral and centroparietal Pe peak amplitude was associated with a higher likelihood of reporting any substance use in the last 12 months (OR =.90, CI[.84 −.96], *p* = .002, *p*_FDR_ =.01; OR =.89, CI[.82–.96], *p* = .002, *p*_FDR_ =.01), as well as with reporting nicotine/tobacco use specifically (OR =.90, CI[.84 −.97], *p* = .004, *p*_FDR_ =.01; OR =.87, CI[.81–.95], *p* = .001, *p*_FDR_ =.01). However, this association disappeared when adjusting for sex and age.

## 4 Discussion

The present study addressed the relationship between ERN and Pe parameters and behavioral parameters (stop accuracy, go accuracy, SSRT, and PES) on the one hand, and adolescent/emerging adult substance use and other externalizing problems on the other hand. We found an association between reduced and delayed frontocentral (but not centroparietal) Pe peak amplitude and decreased task performance, specifically reduced inhibitory control, indicated by longer SSRT. Delayed frontocentral Pe peak latency was associated with lower stop accuracy but not with go accuracy, indicating a possible association between Pe latency and task strategy and/or post-error adjustment, but not with general response accuracy. When accounting for the overlap of neural responses associated with response inhibition and error processing, Pe peak amplitude associations were only observed before a multiple testing correction. This suggests that non-deconvolved Pe parameters may partially reflect other components, such as the P3 component, which is part of an inhibition process, in addition to a Pe that is specifically related to error processing. However, it could also mean that inhibition and error processing potentials are so tightly interlocked that it is not possible to deconvolve Pe and P3.

The analyses revealed no other significant associations between ERN/Pe parameters and task performance, substance use or externalizing problems, and no significant age interactions for these associations. In this section, we discuss various explanations for these findings.

In the present study, larger and earlier frontocentral Pe amplitudes were related to better task performance, which seems in line with studies by e.g. Schroder et al. (2020) and Rueda-Delgado et al. (2021) in adult samples. A link between larger Pe amplitudes and increased post-error accuracy has also been reported (Carp and Compton, 2009). In a late childhood/adolescent sample (ages 8–19 years), no association was reported between Pe amplitude and accuracy and reaction time during a Flanker task, but stronger Pe difference waves – calculated by extracting correct from incorrect trials – were associated with higher accuracy (Overbye et al., 2019). This has been reported in children (ages 5–7 years) as well (Torpey et al., 2012). Taken together, these results indicate that the Pe reflects an important process of behavioral adjustment in order to optimize performance, independent of age.

At the same time, however, we observed no association between ERN parameters and task performance, also after accounting for overlap of neural responses. These null findings are in line with Fiehler et al. (2005), Rodriguez-Fornells et al. (2002), Weinberg et al. (2010), and others (for a review see Gehring et al., 2012), who reported no associations between ERN amplitude and latency and overall task accuracy. Furthermore, null findings have also been reported on the link between ERN parameters and post-error slowing (Buzzell et al., 2017, Dudschig and Jentzsch, 2009, Gehring and Fencsik, 2001, Hajcak et al., 2003, Larson et al., 2016, Riesel et al., 2011). Thus, it has been contested whether ERN parameters and behavioral adjustment are related at all. It has been argued that post-error adjustment – possibly resulting in better task performance - requires conscious error detection (Nieuwenhuis et al., 2001). As the ERN is generally thought to reflect subconscious processing of an error (Endrass et al., 2007, O'Connell et al., 2007), the Pe might be a better indicator of behavior adjustment following errors, considering its assumed role in conscious evaluation and the subjective motivational significance of errors (Overbeek et al., 2005).

An alternative explanation for our null findings might be that ERN parameters rather reflect behavioral adjustment in trials directly following an error, which might not be adequately captured in outcomes such as overall accuracy and average post-error slowing. Furthermore, large trial-by-trial variability in the shape of the ERN might lead to an attenuated ERN peak amplitude when averaged across many trials. Indeed, on a single-trial basis, ERN amplitude has been associated with post-error slowing (Cavanagh and Shackman, 2015, Fischer et al., 2016), and to a lesser extent with post-error accuracy (Carp and Compton, 2009, Kalfaoglu et al., 2018, Themanson et al., 2012). Post-error accuracy has been proposed as a better indicator of post-error behavioral adjustment, as the adaptive goal of post-error slowing depends on task-specific factors such as time between trials and cognitive demands (Danielmeier and Ullsperger, 2011, Schroder and Infantolino, 2013). This raises the question whether behavioral adjustment reflected by Pe parameters is also captured differently in single-trial analyses. Although literature on this topic is limited, studies using single-trial analyses on Pe parameters and task performance have shown an association between Pe amplitude and post-error accuracy (Carp and Compton, 2009), as well as no association between Pe and post-error slowing (Fischer et al., 2016).

Regarding the possible links between ERN and Pe and substance use and externalizing problems, we only observed a trend for a relationship between a delayed frontocentral Pe peak and more externalizing behavior, and for a relationship between smaller frontocentral and centroparietal Pe peak amplitude and a higher CRAFFT score. After multiple testing correction, no significant associations were observed between ERN/Pe parameters and substance use problems or externalizing problems. Earlier studies in non-clinical adolescent populations reported that reduced Pe amplitudes, but not ERN amplitudes, were related to alcohol use (Franken et al., 2017) and inattention traits (Herrmann et al., 2009). At the same time, reduced ERN amplitudes have been consistently observed in individuals with externalizing disorders (Lutz, Kok, et al., 2021) and SUD (Luijten et al., 2014). A possible explanation for this discrepancy involves differences in ERN and Pe function. In addition to its assumed role in conscious error processing, the affective processing hypothesis suggests that the Pe can reflect a motivational component (Overbeek et al., 2005, Ridderinkhof et al., 2009). In other words, the Pe might reflect how much an individual ‘cares’ about the error made. In that way, Pe parameters might reflect part of a motivational-reward pathway that has been associated with the development of both SUD (Bjork et al., 2012, Blum et al., 2000) and other externalizing disorders (Sonuga-Barke, 2005).

However, it is important to take into account that externalizing problems, and especially substance-related risks and problems, were limited in the current sample. Among the general youth population in Norway, fifty percent of 15/16-year olds report having consumed alcohol in the last 12 months, with an average of 7 drinking episodes within this time interval (Bye and Skretting, 2017, Molinaro et al., 2020). In contrast, in the current sample, despite having a substantially older mean age of 18 years (*SD* = 2.5), only 65 % of participants used any alcohol in the last 12 months. Concurrently, substance use problems measured by the CRAFFT questionnaire were on average low, with 52 % of the sample having a sum score of zero (see Table 1). Furthermore, since some participants of the current sample were under 15 years old (see Table 1), and thus below the average age of substance use initiation in Norway (Bye and Skretting, 2017), we were unable to get a full picture of their substance-related risks and problems throughout adolescence. This may have attenuated associations between ERN/Pe parameters and substance use behavior. Finally, 73.7 % of the sample were women. Considering that the prevalence of both substance use problems (McHugh et al., 2018) and externalizing disorders (Mayes et al., 2020) is higher in men, this skewed distribution could limit generalizability of our findings, and hindered our ability to explore sex-specific effects. Other limitations include the use of self-report questionnaires, which might increase the risk for social desirability bias or underreporting due to individuals’ limited insight into the extent of their substance use or externalizing behaviors. Furthermore, the recruitment method of the current study may have introduced biases in sample composition (e.g. regarding socioeconomic status (SES)) which could not be mitigated within the scope of the available data, limiting generalizability of findings. When considering task-related limitations, it must be taken into account that the stop-signal task assesses a specific type of response inhibition (i.e., inhibiting a prepotent response; Verbruggen et al., 2019), which might not fully capture the error processing aspects that are associated with substance use behavior. Furthermore, in paradigms such as the present one, where stimulus- and response-locked ERPs are generated in close temporal proximity, there is a risk of overlapping ERPs. Although we attempted to account for this overlap with deconvolution, it remains challenging to disentangle inhibition and error processing processes due to their tight coupling and potential interaction.

Strengths of the present study include its sample size (*N* = 143) and using a stop-signal task with a sufficient number of stop trials to achieve a reliable SSRT estimate, while maintaining a sufficiently low stop trial probability, resulting in an average of 38.4 error epochs per individual. It must be noted however, that the exclusion of participants with an insufficient number of error epochs, due either to high task proficiency or a cautious response style, may have reduced the representativeness of the findings. Another strength of the study is the use of the CRAFFT questionnaire, which is sensitive to detect risk for future substance use disorder, extending beyond the more commonly used measures of quantity and frequency of use (Shenoi et al., 2019), even in nonclinical samples. Finally, our study accentuates the importance of implementation of multiple testing correction methods such as FDR adjustment. Especially in samples with limited variability in variables of interest, which is often the case for measures of externalizing problems in population-based samples, there is an increased risk for the occurrence of spurious findings. Applying an FDR correction increases the likelihood that significant findings reflect real associations between variables rather than chance findings (Lindquist and Mejia, 2015).

Future research using single-trial analyses, capturing trial-by-trial variability, could add more insight into the relationship between error processing ERPs and performance on response inhibition tasks. Moreover, time-frequency analyses could be utilized to better understand the oscillatory dynamics underlying task performance, that might not be captured by traditional ERP methods. Furthermore, future studies should investigate the robustness of associations between ERN/Pe parameters and behavioral outcomes in light of SES factors. Finally, longitudinal studies are needed to determine whether adolescent ERN/Pe parameters are associated with response inhibition and externalizing problems later in life, offering valuable insights into early detection or prevention strategies.

In summary, the current study indicates that frontocentral Pe peak amplitude and latency are an indicator of response inhibition task performance in an adolescent and emerging adulthood sample, but that ERN/Pe parameters do not seem to be related to subclinical externalizing behavior and substance-related risks and problems. Given the relatively limited literature on the Pe in relationship to response inhibition and externalizing behavior, particularly in terms of its latency, further research is needed to assess robustness of our findings within adolescent populations.

## 5 Financial support

The current study was supported by the Ter Meulen Grant from the Royal Netherlands Academy of Arts and Sciences [grant number TMB403] (OB), Stichting Volksbond Rotterdam (IF & HM), the Netherlands Organization for Health Research and Development [Aspasia grant No.015.016.056] (HM), the European Research Council (10.13039/100010663ERC) under the European Union’s Horizon 2020 research and innovation program (grant agreement No 802998), the Research Council of Norway (#223273, #288083, #323951), and the South-Eastern Norway Regional Health Authority (2019101, #2021040, #2021070, #2023012, #500189). This work was performed on the Tjenester for Sensitive Data (TSD) facilities, owned by the University of Oslo, operated and developed by the TSD service group at the University of Oslo, IT-Department (USIT) and on resources provided by UNINETT Sigma2—the National Infrastructure for High Performance Computing and Data Storage in Norway. The funders had no role in the design and conduct of the study or the writing of the report.

## CRediT authorship contribution statement

**Olga D. Boer:** Conceptualization, Data curation, Formal analysis, Methodology, Project administration, Visualization, Writing – original draft. **Thea Wiker:** Data curation, Formal analysis, Investigation, Methodology, Writing – review & editing. **Shervin H. Bukhari:** Data curation, Formal analysis, Writing – review & editing. **Rikka Kjelkenes:** Investigation, Writing – review & editing. **Clara M. F. Timpe:** Data curation, Investigation, Writing – review & editing. **Irene Voldsbekk:** Investigation, Writing – review & editing. **Knut Skaug:** Data curation. **Rune Boen:** Methodology, Writing – review & editing. **Valerie Karl:** Investigation, Writing – review & editing. **Torgeir Moberget:** Conceptualization, Writing – review & editing. **Lars T. Westlye:** Conceptualization, Project administration, Writing – review & editing. **Ingmar H. A. Franken:** Methodology, Writing – review & editing. **Hanan El Marroun:** Writing – review & editing. **Rene J. Huster:** Conceptualization, Data curation, Methodology, Writing – review & editing. **Christian K. Tamnes:** Conceptualization, Methodology, Project administration, Supervision, Writing – review & editing.

## Declaration of Generative AI and AI-assisted technologies in the writing process

During the preparation of this work the authors used ChatGPT (OpenAI) in order to explore alternative wording or phrasing for segments of their own-written sentences. After using this tool, the authors reviewed and edited the content as needed and take full responsibility for the content of the publication.

## Declaration of Competing Interest

One of the authors (C.K. Tamnes) is an Editorial Board Member for *Developmental Cognitive Neuroscience* and was not involved in the editorial review or the decision to publish this article.

## Data Availability

The data that has been used is confidential.
