# Supplementary material for: Neural markers of error processing relate to task performance, but not to substance-related risks and problems and externalizing problems in adolescence and emerging adulthood
Source: Dev Cogn Neurosci. 2024 Dec 24;71:101500. doi: 10.1016/j.dcn.2024.101500 (PMC11732202; doi:10.1016/j.dcn.2024.101500)
Supplement: Supplementary material [file mmc1.docx]

**Supplemental Figure 1**

**
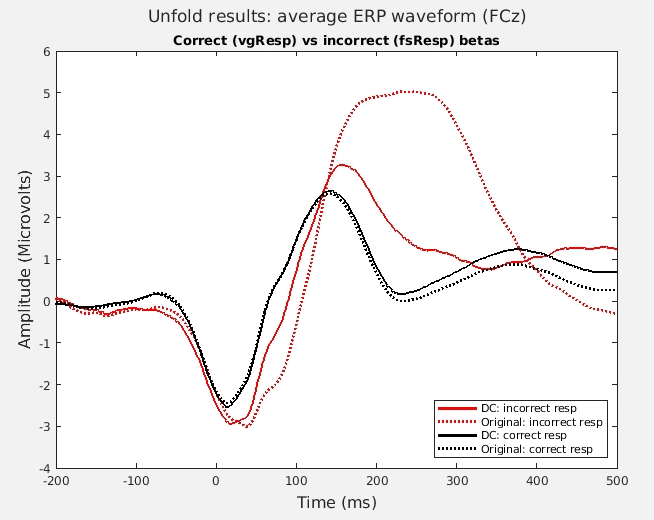
***Grand average response-locked (response at time = 0 ms) event-related potentials for correct (black) and incorrect (red) responses in a stop-signal task, at FCz of N = 142 participants. The continuous lines represent the ERP waveform after deconvolution was performed to account for the potential overlap of neural responses and to isolate event-specific neural activity (Ehinger & Dimigen, 2019).*
